# Hypoglycemic and Hypotensive Activity of a Root Extract of *Smilax aristolochiifolia*, Standardized on N-*trans*-Feruloyl-Tyramine

**DOI:** 10.3390/molecules190811366

**Published:** 2014-07-31

**Authors:** Carol Arely Botello Amaro, Manasés González-Cortazar, Maribel Herrera-Ruiz, Rubén Román-Ramos, Lucia Aguilar-Santamaría, Jaime Tortoriello, Enrique Jiménez-Ferrer

**Affiliations:** 1 Centro de Investigación Biomédica del Sur, Instituto Mexicano del Seguro Social (IMSS), Argentina No. 1, C.P. 62790 Xochitepec, Morelos, Mexico; E-Mails: carolarely@hotmail.com (C.A.B.A.); gmanases@hotmail.com (M.G.-C.); cibis_herj@yahoo.com.mx (M.H.-R.); llasmusic@hotmail.com (L.A.-S.); jtortora2@yahoo.es (J.T.); 2 Programa de Doctorado de Ciencias Biológicas y de la Salud, Universidad Autónoma Metropolitana-Iztapalapa, Av. San Rafael Atlixco No. 186, Col. Vicentina C.P. 09340, Iztapalapa, México D.F., Mexico; 3 Departamento de Farmacología, Universidad Autónoma Metropolitana-Iztapalapa, Av. San Rafael Atlixco No. 186, Col. Vicentina C.P. 09340, Iztapalapa, México D.F., Mexico; E-Mail: rrr@xanum.uam.mx

**Keywords:** N-*trans*-feruloyl-tyramine, *Smilax aristolochiifolia* Smilacaceae, metabolic syndrome

## Abstract

The metabolic syndrome (MS) is a condition consisting of various metabolic abnormalities that are risk factors for developing kidney failure, cardiovascular, vascular and cerebrovascular diseases, among others. The prevalence of this syndrome shows a marked increase. The aim of this study was to investigate the pharmacological effect of *Smilax aristolochiifolia* root on some components of MS and obtain some of the active principle using chromatographic techniques. The compound isolated was N-*trans*-feruloyl tyramine NTF (1), and its structure was determined by spectroscopic and spectrometric analyses. The whole extract and the standardized fractions were able to control the weight gain around 30%; the fraction rich in NTF was able to decrease the hypertriglyceridemia by 60%. The insulin resistance decreased by approximately 40%; the same happened with blood pressure, since the values of systolic and diastolic pressure fell on average 31% and 37% respectively, to levels comparable to normal value. The treatment also had an immunomodulatory effect on the low-grade inflammation associated with obesity, since it significantly decreased the relative production of pro-inflammatory cytokines regarding anti-inflammatory cytokines, both kidney and adipose tissue. Therefore it can be concluded that the extract and fractions of *Smilax aristolochiifolia* root with NTF are useful to counteract some symptoms of MS in animal models.

## 1. Introduction

The metabolic syndrome (MS) is a complex and heterogeneous clinical entity that includes obesity, hyperglycemia, hypertriglyceridemia and elevated blood pressure [1]. Its expression is strongly influenced by environmental, social, cultural and economic factors [2]. The parallel increase of the frequency of the metabolic syndrome and obesity is a worldwide phenomenon, particularly important in México [3]. These pathologies are major risk factors for developing coronary heart disease, heart failure and cerebrovascular atherosclerosis [1], which are the leading cause of death in the country [4]. Obesity appears to be one of the most important factors for triggering MS [5]. By controlling the pathophysiological alterations that are included in the MS, the morbidity and mortality of this syndrome is reduced [6].

It has been shown that mice of the C57/BL/6 strain respond in a relatively short time to a high‑caloric diet (saturated fat and carbohydrates) developing several parameters of MS (obesity, hypertension, insulin resistance and hypertriglyceridemia) [7]. The morbidly obese condition generates a state of low-grade inflammation, which differs from the states of chronic and acute inflammation, and alters the levels of pro-inflammatory cytokines like IL-1β, IL-6 and TNFα [8] regarding anti‑inflammatory cytokines like IL-10. In addition, angiotensin II (AGII) was administered at a low dose to produce endothelial damage, since this produces an inflammatory response, and it was maintained throughout the period of time in which the drug was administered [9].

There are reports in the literature of some chemical compounds in the genus *Smilax*, such as steroidal saponins with anti-inflammatory properties [10], flavonoids with anti-oxidant [11] and anti‑fungal [12] activity. The root of *Smilax aristolochiifolia* Miller (Smilacaceae) is widely used in the food industry as a flavoring; its medicinal use is indicated for weight loss, as diuretic, anti-diabetic, sudorific and depurative [13]. The aim of this study was to evaluate the pharmacological effect of extracts of *S. aristolochiifolia* on some degenerative components of MS and the underlying inflammatory condition, in a model of MS induced by high-caloric diet and chronic administration of AGII.

## 2. Results and Discussion

### 2.1. Chemical Characterization

**Figure 1:**
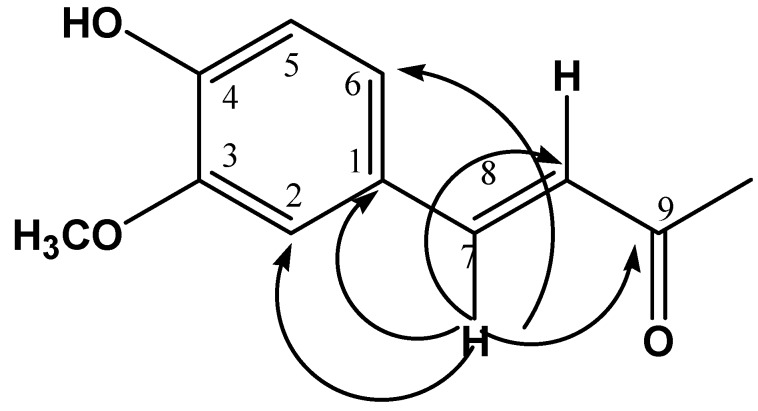
HMBC correlations of H-7.

Chromatographic fractionation of the F2, allowed us to obtain compound **1** as a yellow precipitate, soluble in methanol, with a melting point of 74–75 °C. In the UV-vis spectrum, the compound showed the following λ_max_: 220, 293, and 318 nm. The ^1^H-NMR spectrum showed four systems: an aromatic ABX system [δ 7.10 (1H, d, *J* = 2 Hz), 6.79 (1H, d, *J* = 8.2 Hz) and 7.0 (1H, dd, *J* = 2, 8.2 Hz)] assigned a H-2, H-5 and H-6 respectively; another aromatic A_2_B_2_ system [δ 7.05 (2H, d, *J* = 6.8 Hz) and 6.72 (2H, d, *J* = 7.2 Hz)] assigned H-2', H-6' and H-3', H-5' respectively; a *trans* double bond AB system [δ 7.43 (1H, d, *J* = 15.6 Hz) and 6.40 (1H, d, *J* = 15.6 Hz)] assigned a H-7 and H-8 respectively, and a AB system [δ 2.75 (2H, dd, *J* = 7.1, 7.5 Hz) and 3.46 (2H, dd, *J* = 7.2, 6.5 Hz)] assigned a H-7' and H-8' respectively. According to a HMBC experiment, H-7 (δ 7.43) correlated at two and three bonds with five carbon signals at δ 128.39, 111.6, 123.33, 118.8 and 169.28 which are assigned to C-1, C-2, C-6, C-8 and C-9 respectively, indicating the presence of a *trans*-feruloyl group (see Figure 1).

**Figure 2:**
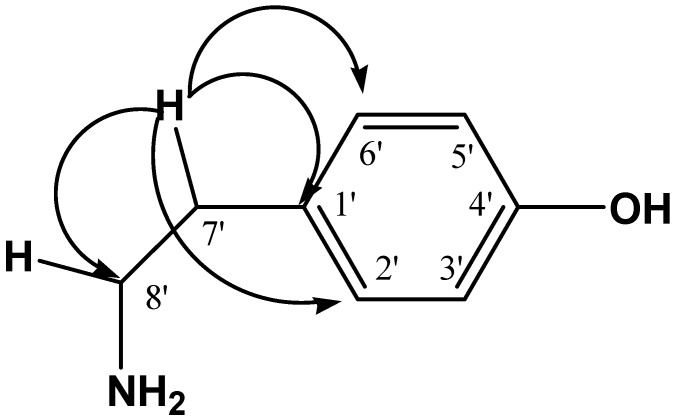
HMBC correlations of H-7'.

On the other hand the signal of H-7' (δ 2.75) correlated to J_2-3_ with four signals ^13^C in δ 131.41, 130.85, 130.85 and 42.66 assigned to C-1', C-2', C-6' and C-8' respectively, indicates the presence of ethyl benzene (see Figure 2).

**Figure 3:**
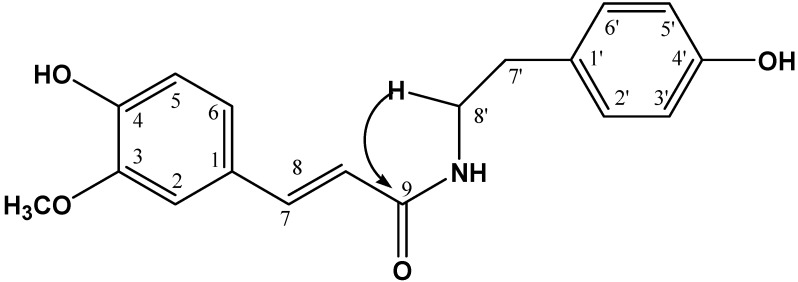
HMBC correlations of H-8'.

Additionally, the signal H-8' (δ 3.46) is attached to a group containing a nitrogen atom for its chemical shift. This allowed us to determine the presence of a terminal amine group, forming the skeleton of tyramine. This group is attached to the N-*trans*-feruloyl moiety, according to the observed coupling of H-8' to J_2-3_ with the signal of the C-9 carbonyl group (169.28) of an amide ester (see Figure 3).

**Figure 4:**
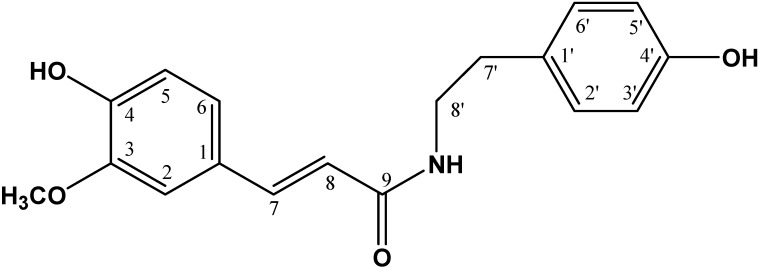
Chemical structure of N-*trans*-feruloyltyramine.

In conformity with NMR analysis data and comparison of the literature data [14], the chemical structure was established as N-*trans*-feruloyltyramine or NTF (**1**) (see Figure 4).

The mass spectrum gave a molecular ion of m/z 314 [M + H]^+^, 192 [M-ethylbenzene], 177 [M-ethylaminobenzene]. This compound has not been reported previously for *S. aristolochiifolia*. However, phenylethylcinnamide has been isolated from other species, and possesses activities such as antioxidative [15,16], α-glucosidase inhibitor [17] and antiarrhythmic [18]. It was used for the standardization of the extract of *S.* *aristolochiifolia* by HPLC and the content of NTF in each sample of mg/g of extract was: acetone extract (AceSa, 2.77), F1 (2.63), F2 (60.22), F3 (8.76).

### 2.2. Chemical Analysis

**Figure 5:**
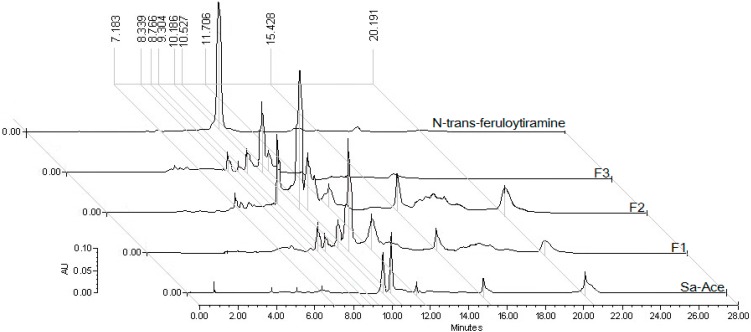
Chromatographic profile by HPLC of different samples from *Smilax* *aristolochiifolia* (AceSa, F1, F2 and F3, and N-*trans*-feruloyl-tyramine which had a RT = 10.186 min).

The analysis by HPLC showed a different chromatographic profile of samples from *S. aristolochiifolia* (Figure 5). The retention time (RT) for NTF is 10.18 min, and the AUC for the peak of this compound in each sample indicated that the fraction F2 contains a higher concentration of NTF than AceSa, F1 or F3. The calibration curve allowed us to calculate the doses of NTF administered in each treatment. AceSa was administered at a dose of 69.25 μg/kg, F1 65.75 μg/kg, F2 600 μg/kg, and F3 219 μg/kg (see Figure 5).

### 2.3. Pharmacological Characterization

**Figure 6:**
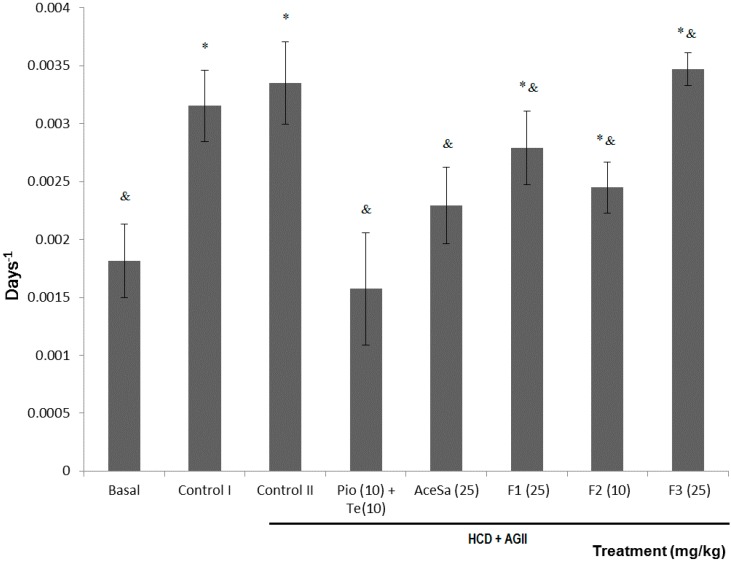
Effect of different treatments on the growth rate of C57/BL-6 mice exposed to hypercaloric diet and chronic angiotensin II (control II): acetone extract of *Smilax aristolochiifolia* (AceSa), fractions F1, F2 and F3 from AceSa, pioglitazone and telmisartan (Pio + Tel). Control I = hypercaloric diet. ANOVA followed by Tukey *post‑test* (mean ± SD, *n* = 10); * *p* < 0.05 (in comparison with the basal group), ^&^ *p* < 0.05 (in comparison with control II).

The effect of the administration of a high-caloric diet (HCD, control I group) for 20 weeks was compared against the effect of a regular diet (basal group) and against the negative control of the experiment (HCD + AGII, control II group). In both cases, a twofold increase was observed in growth rate values (which corresponds to the slope of the logarithmic phase of the change of weight with respect to time in days) compared to the values observed in the basal group; these data were significantly different from those of the basal group (* *p* < 0.05) (see Figure 6).

The treatments with AceSa extract and positive control (Pio + Tel) showed no significant change (*p* > 0.05) compared to baseline conditions. But when these groups were compared with the damage group (control II) it is showed a significant decrease (^&^ *p <* 0.05, Figure 6). Although the effect of F1, F2 and F3 was lower than AceSa and it was significantly different from the basal group (* *p* < 0.05), these treatments also induced a statistically decrement of the growth velocity curve with respect to the control II group (^&^ *p* < 0.05).

**Figure 7:**
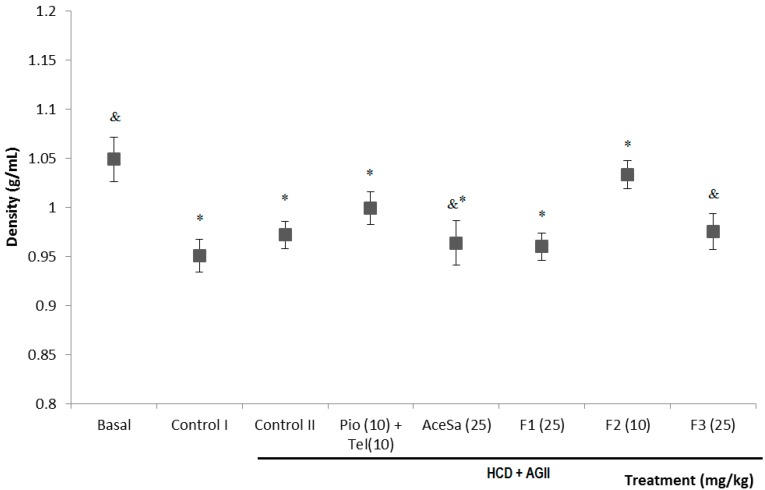
Effect of different treatments on the body density of C57/BL-6 mice exposed to hypercaloric diet and chronic angiotensin II (control II): acetone extract of *Smilax aristolochiifolia* (AceSa), fractions F1, F2 and F3 from AceSa, pioglitazone and telmisartan (Pio + Tel). Control I = hypercaloric diet. ANOVA followed by Tukey *post‑test* (mean ± SD, *n* = 10); * *p* < 0.05 (in comparison with the basal group), ^&^ *p* < 0.05 (in comparison with control II).

Mice fed with a regular diet (basal) showed an increment dependent on the increase of lean tissue, reaching a value of body density of 1.05 ± 0.02 g/mL (Figure 7). In the groups with HCD or HCD + AGII, control group I and II respectively, was observed a significant decrement of the body density with respect to the basal group (* *p* < 0.05); this effect results from the increase in weight that can be calculated from the values in the growth rate (Figure 6).

The treatments AceSa, F1 and F3 from *S.* *aristolochiifolia* did not prevent the effect of HCD and AGII on the body density. Data from these groups were significantly different from the basal (Figure 7, * *p* < 0.05), but not from the damage group (Figure 7, * *p* > 0.05). However, the fraction F2 and the positive control group (Pio + Tel) were able to inhibit the decrement of body density induced with HCD and AGII. This effect was different to the damage group (HCD + AGII, *^&^ p* <0.05), but statistically similar to the basal (*p* > 0.05).

**Figure 8:**
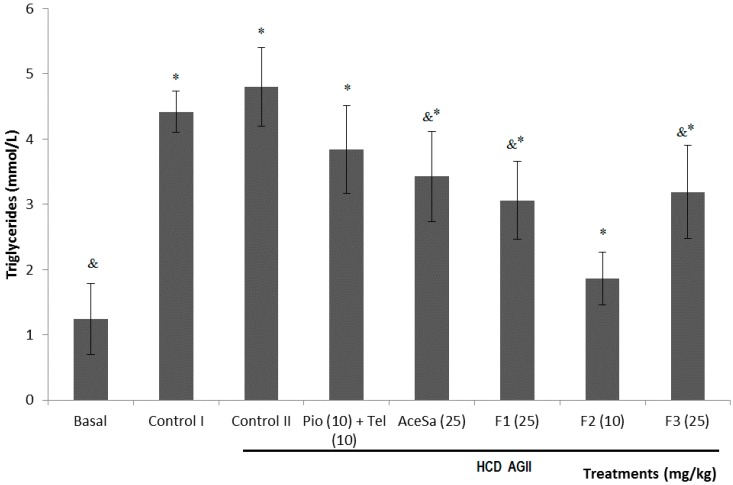
Effect of different treatments on the serum concentration of triglycerides of C57/BL-6 mice exposed to hypercaloric diet and chronic angiotensin II (control II): acetone extract of *Smilax aristolochiifolia* (AceSa), fractions F1, F2 and F3 from AceSA, pioglitazone and telmisartan (Pio + Tel). Control I = hypercaloric diet. ANOVA followed by Tukey *post-test* (mean ± SD, *n* = 10); * *p* < 0.05 (in comparison with the basal group), ^&^ *p* < 0.05 (in comparison with control II).

Hypertriglyceridemia is a cardinal alteration of MS associated with dyslipidemia. In this work, the administration of HCD and HCD + AGII caused a significant increase (* *p* < 0.05) in the concentration of plasma triglycerides, which increased in respect to the basal group up to 3 and 4 times, respectively. Although the treatments from *S.* *aristolochiifolia* were higher and statistically different to basal group (* *p* < 0.05), they provoked a statistical significant diminish of the triglyceride serum concentration in regard to the damage group with HCD + AG II (^&^ *p* < 0.05), and it was worth noting that the group of mice treated with F2 responded better, given that the triglycerides were lower than HCD + AG II but statistically equal to basal (Figure 8), while the group with Pio + Tel, did not diminish this parameter in respect to the control II group (*p* > 0.05, Figure 8).

**Figure 9:**
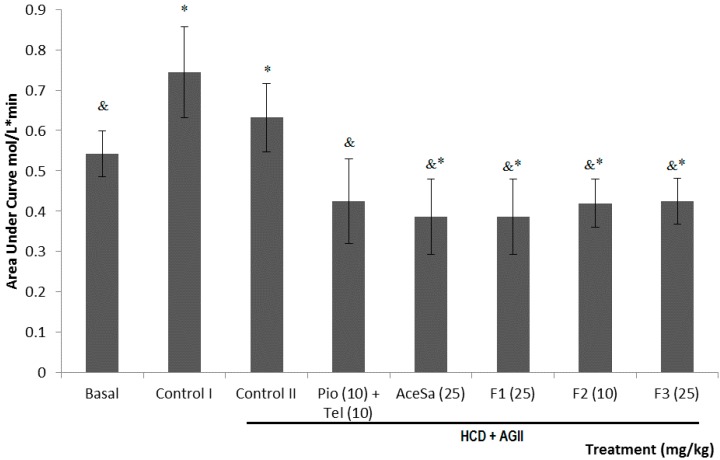
Effect of different treatments on the insulin resistance (measured as the area under the curve) of C57/BL-6 mice exposed to hypercaloric diet and chronic angiotensin II (control II): acetone extract of *Smilax aristolochiifolia* (AceSa), fractions F1, F2 and F3 from AceSA, pioglitazone and telmisartan (Pio + Tel). Control I = hypercaloric diet. ANOVA followed by Tukey *post-test* (mean ± SD, *n* = 10); * *p* < 0.05 (in comparison with the basal group), ^&^ *p* < 0.05 (in comparison with control II).

Another component of MS is the insulin resistance that leads to type 2 diabetes mellitus. This parameter is shown as the area under the curve (AUC) of plasma glucose concentration with the administration of a dose of insulin. The animals exposed to both HCD and HCD + AGII showed a significant increment value of the AUC respect to basal (* *p* < 0.05, Figure 9). All treatments from *S.* *aristolochiaefolia* decreased the insulin resistance parameter by decreasing the value of AUC data, that were statistically different to the group with damage (^&^ *p* < 0.05, Figure 9). The positive control treatment (Pio + Tel) was the best to cause a decrement of this parameter (^&^ *p* < 0.05), even with a greater reduction than the observed for the basal (* *p* < 0.05).

**Figure 10:**
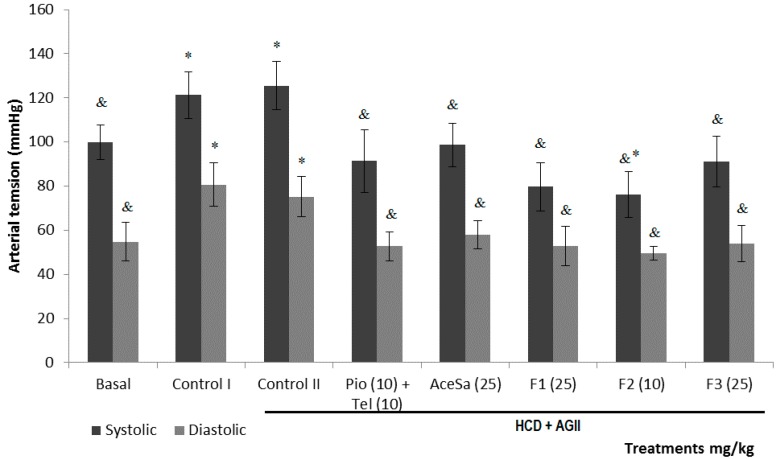
Effect of different treatments on systolic and diastolic blood pressure (SBP and DBP) of C57/BL-6 mice exposed to hypercaloric diet and chronic angiotensin II (controlII): acetone extract of *Smilax aristolochiifolia* (AceSa), fractions F1, F2 and F3 from AceSA, pioglitazone and telmisartan (Pio + Tel). Control I = hypercaloric diet. ANOVA followed by Tukey *post-test* (mean ± SD, *n* = 10); * *p* < 0.05 (in comparison with the basal group), ^&^ *p* < 0.05 (in comparison with control II).

When the diastolic (DBP) and systolic (SBP) blood pressure were measured, it was observed that groups of mice with HCD or HCD + AGII showed a substantial and statistically significant increase in both, in comparison to the basal situation (* *p* < 0.05, Figure 10). This increment was controlled with the administration of extract (AceSa) and fractions (F1, F2 and F3) from *S. aristolichiifolia* to different animal groups; therefore a decrease of SBP and DBP could be observed (^&^ *p* < 0.05, Figure 10).

**Figure 11:**
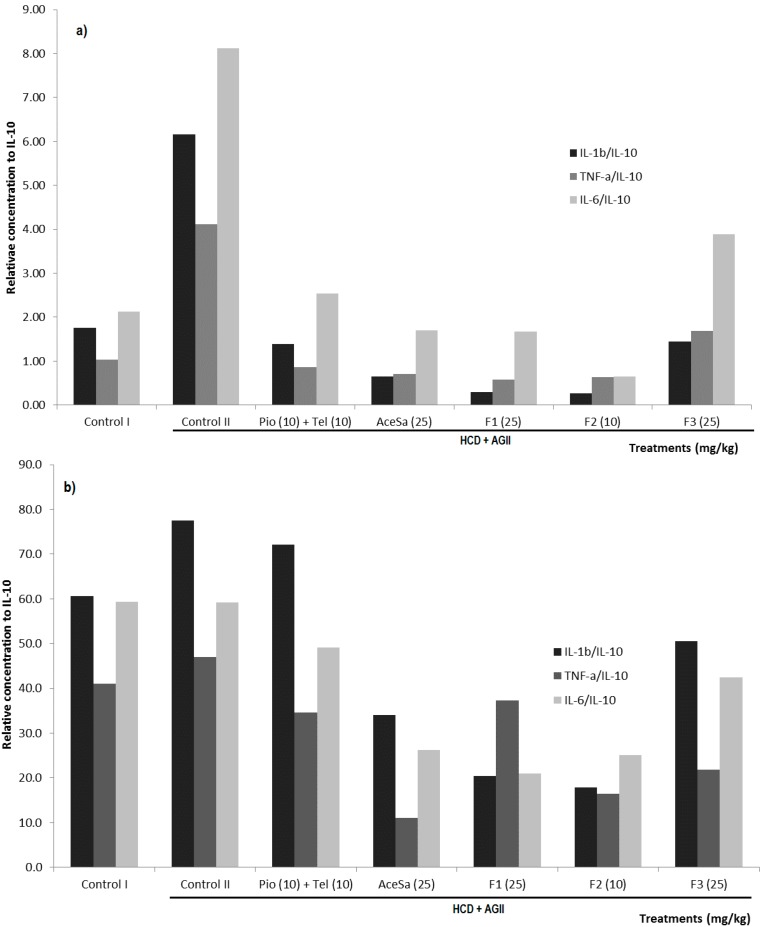
Effect of different treatments on concentration of cytokines IL-1β, TNF-α and IL-6 with respect to the concentration of IL-10, in kidney (**a**) and fat tissue (**b**) of C57/BL-6 mice exposed to hypercaloric diet and chronic angiotensin II (control II): acetone extract of *Smilax* *aristolochiifolia* (AceSa), fractions F1, F2 and F3 from AceSA, pioglitazone and telmisartan (Pio + Tel). Control I = hypercaloric diet.

The levels of cytokines were quantified in kidney and adipose tissue from animals with different experimental conditions associated with MS. The kidney administration of treatments and HCD or HCD + AGII established an inflammatory environment, in comparison to the basal conditions (Figure 11a). When this inflammatory process was evaluated in the groups of mice with extract or fractions from *S.* *aristolochiifolia* (Figure 11a), a decrease of the pro-inflammatory conditions was generally observed, in the same way as it happened with the positive control treatment (Pio + Tel).

In regard to the response of the adipose tissue that can be observed in Figure 11b, both treatments HCD and HCD + AGII had a similar effect. Positive control treatment (Pio + Tel) was not able to decrease the pro-inflammatory response in adipose tissue (Figure 11b), in contrast to mice with treatments from *S.* *aristolochiifolia* which generally showed decreased tissue pro-inflammatory responses, although fraction F3 did so to a lesser extent (Figure 11b).

In this work, a high-caloric diet was administered to C57BL6 mice, control group I, increasing their weight gain and generating damage associated with MS: overweight, hyperglycemia, hypertension, and hypertriglyceridemia. These alterations may eventually cause vascular damage, but not necessarily because of the short period of exposure time. Due to the above control group II that was used, potential vascular disorders associated with SM ailments were exacerbated with the chronic administration of the potent vasoconstrictor AG II. The latter caused the establishment of organic damage to vasculature, through the oxidative stress and inflammatory processes in endothelial cells.

The increase in glucose concentration, triglycerides and chronic hypertension associated with obesity and diabetes is the major pathological factor for endothelial dysfunction, which is a key element of the mechanisms underlying vascular disease [19]. It is also a predictor of clinical events that have a high rate of comorbidity, such as cerebral vascular accidents [20,21], mainly due to the fact that endothelial cells play an important role in regulating vascular structure and function [19].

Obesity is a risk factor for the development of hypertension and dyslipidemia. Weight gain induced by HCD produces an increase in the plasma concentrations of AG II, which raises systolic blood pressure in male mice [22]. Adipocytes, the main cells of adipose tissue, are capable of expressing angiotensinogen and angiotensin peptides [22].

In humans, AG II produced by adipocytes seems to inhibit the differentiation of adipocyte precursors, decreasing the percentage of small adipocytes sensitive to insulin. Thus, the storage capacity of lipids in the adipose tissue is reduced, and the triglycerides can accumulate in the liver and in the skeletal muscle, contributing to insulin resistance [23]. Besides, the steady increase in the level of AGII producing a prolonged vascular constriction induces release of aldosterone, retention of sodium and water and increase of fluids, ultimately leading to a state of hypertension [24]. Thus, a chronic imbalance of AG II regulation induces chronic hypertension and vascular damage, as well as dyslipidemia and hyperglycemia; this was reproduced in the model used in this work with an HCD and AG II diet. Prolonged hyperglycemia and insulin resistance result in hyperinsulinemia; it up-regulates the components of the renin-angiotensin system and sharpens the pro-inflammatory and pro-fibrotic condition mediated by AG II, which is associated with macrovascular complications of diabetes. The imbalance of RAS due to the increase of AG II in tissues such as kidney, liver and adipose tissue by hyperglycemia may contribute to hypertension associated with MS and to the organ damage observed in diabetes mellitus II [23].

The data obtained in this work showed for the first time the effect of *S. aristolochiifolia* on an injury model associated with MS. The previous pharmacological use of this plant indicated only the diuretic effect [14]. There are other works which have shown that species of the same genus induced a beneficial effect on different alterations associated to MS. For example, *S. officinalis*, inhibits α-glucosidase enzymes and possesses an antioxidant effect [25]; *S. canariensis* has a diuretic effect [26]; *S. aspera* and *S. china* Linn decrease the plantar inflammation induced by carrageenan [27,28]. The latter possesses the compound sieboldogenin that inhibits the activity of lipoxygenase [28]. Glycosides (smilasides G-L) from *S. bracteata* have scavenger activity against DPPH radicals [29] and *S. glabra* has shown a hypoglycemic effect in a test of insulin tolerance in mice [30].

In the present study, the administration of the AceSa extract and fractions F1, F2 and F3 from *S. aristolichiifolia* were an effective treatment to control the major alterations associated with the MS, in C57BL6 mice with damage induced by HCD and chronic administration of AG II. This is the case of the AceSa extract, which counteracted the effect of the HCD diet and AG II on the growth curve (corroborated by the decrease of body density), and kept the animals under conditions similar to those of the baseline group. This effect was also observed in the group that received the combination of drugs pioglitazone and telmisartan. This is likely due to the presence of phenolic compounds in AceSa extract, since they have the capacity of inhibiting α-glucosidase enzymes, which are a therapeutic target for the treatment of obesity in humans [17]. Furthermore, triglyceride levels in mice with a HCD + AGII diet were higher than in animals that received only an HCD diet. This parameter decreased significantly in all groups that received some treatment from *S. aristolochiifolia* (AceSa, Fi, F2 and F3), in comparison with the control II group. Although the results with F2 are worth noting, this treatment was better than all groups including the treatment with pioglitazone and telmisartan. Serum glucose levels, hypertension, and the local concentration of pro-inflammatory cytokines induced by HCD + AGII diet (control II) also decreased in the animals that received any treatment of *S. aristolochiifolia*.

The chemical analysis indicated that all *S. aristolochiifolia* preparations used for the treatment of animals with MS contained different concentrations of isolated compound NTF; the highest level of this substance is in fraction F2. Then, all the pharmacological effects against MS in mice could be attributed mainly to the presence of NTF, because F2 possesses the major effect and the concentration of this compound is higher in it. This does not rule out the possibility of pharmacological interaction with other active principles, and that the pharmacological effect observed could be the result of different activities related to the complex composition of the fraction.

It is worth noting that the effects of different treatments of *S. aristolochiifolia* could be due to NTF because it has been described as a noncompetitive inhibitor of α-glucosidases, which explains its anti‑hyperglycemic capacity [17]. Also, it was shown that NTF has an antioxidant capacity through the sequestration of free radicals [15]. On the other hand, NTF counteracts the inflammatory disorders of MS, and a dose-dependent regulation effect on the release of nitric oxide (respiratory burst) stimulated by LPS in a cell culture RAW 264.7 has been demonstrated [31].

However, the most remarkable pharmacological effect of NTF, which might explain part of the effect of the extract of *S. aristolochiifolia* as anti-MS, is that it acts as ligand for the gamma receptor activated by the peroxisome proliferator (PPAR-γ). Therefore, NTF is capable of improving the morbid state of visceral obesity and the conditions resulting from this disease. This occurs because PPAR-γ2 is expressed specifically in adipose tissue and is a master regulator in the differentiation and maturation of adipocytes [32]. It has been shown that ligands for PPAR-γ2, improve the condition of insulin resistance. For all this, the administration of these substances can be an effective treatment for the prevention and/or improvement not only of type 2 diabetes mellitus, but also of the insulin resistance syndrome with hyper-insulinemia, abnormal lipid metabolism, obesity, hypertension and atherosclerotic disease [33].

It is noteworthy that the administrations of synthetic drugs of the type of glitazones (which act on PPAR) have shown adverse side effects, especially cardio-toxicities such as congestive heart failure, arrhythmias and peripheral edema [34]. Nevertheless, the widespread use of the extract of *Smilax aristoliochiifolia* in soft drinks such as “*zarzaparilla*” root beer allows us to assume that NFT might be free of any toxic effects. Furthermore, MS-associated inflammation was monitored by quantifying the concentration in kidney and in adipose tissue of pro-inflammatory cytokines such as IL-6, IL-1β and TNF-α, as well as the anti-inflammatory IL-10.

It was observed that the group with HCD diet had a non-significant increase in the concentration of pro-inflammatory cytokines with respect to the level of IL-10 in the kidney; even so, the injury model fed with HCD and AG II showed a significant increase of these molecules in the kidney, indicating that the chronic administration of AG II induces an exacerbated pro-inflammatory state.

Obesity and hypertension are recognized as diseases with an important inflammatory background; a study showed that hypertensive and obese patients had low levels of IL-10 and high serum concentrations of IL-8, compared with hypertensive subjects with normal weight [35]. The mechanism of AG II-mediated chronic hypertension also activates T cells by AGII, which promotes vascular inflammation and elevated pressure [36]. This explains the essential role described for TNF-α as a blood pressure regulator. Regarding the increase in blood pressure caused by the infusion of AG II in hypertensive rats fed with high salt diet, etanercept (TNF-α inhibitor) retards the progression of hypertension, an effect that is attributed to a reduction of kidney damage, due to a decrease of proteinuria and of the infiltration of macrophages/monocytes [37]. Another study indicates that in knockout mice for TNF-α, the infusion of AG II for two weeks fails to cause hypertension [38].

## 3. Experimental Section

### 3.1. General Procedures

The melting point of the isolated compounds was obtained on Thermo Scientific equipment IA1900 series (Thermo Scientific Inc., Hudson, NH, USA); the results shown are uncorrected. NMR spectra were recorded on Varian INOVA-400 at 400 MHz for ^1^H and ^13^C, and two-dimensional spectroscopy experiments COSY, HSQC, HMBC in CD_3_OD. Chemical shifts are reported in parts per million (ppm) relative to tetramethylsilane (TMS). We used a Hewlett Packard 5985-B and a JEOL-AX 505 HA mass spectrophotomete.

### 3.2. Plant Material

This project was approved by the research committee of the IMSS (Instituto Mexicano del Seguro Social) on June 17, 2010, with registration number R-2010-1701-41. The root of *Smilax aristolochiifolia* was obtained in Monte Blanco (19°23'02.20" N and 96°56'09.60" W at 1773 masl), in the municipality of Teocelo, Veracruz, Mexico. A specimen was identified and deposited for reference in the Herbarium of INAH-Morelos (Medicinal Plant Herbarium of the Institute of Anthropology and History-Morelos). The registration number given was 2053 and the identification was made by Margarita Avilés Flores.

### 3.3. Extraction and Fractionation of the Root of Smilax aristolochiifolia

The dried and ground root (3.88 kg) was extracted three times with acetone (20 L) at 25 °C for 24 h. The acetone extract of *S. aristolochiifolia* (Ace-Sa) was concentrated in a rotary evaporator (Heidolph Laborota 4000, Schwabach, Germany). The Ace-Sa (35 g) was suspended in *n*-hexane (3.0 L) to give 3 g of a soluble fraction and 30.0 g of a precipitate. This precipitate was dissolved in chloroform (1.2 L) to give 6.2 g of a soluble fraction and 16.0 g of insoluble precipitate (called **F1**). The fraction **F1** was dissolved in acetone (2,000 mL) to give 11.5 g of the soluble fraction (called **F2**) and 3.0 g of the insoluble precipitate (called **F3**).

**F2** (11.5 g) was fractionated using silica gel (345 g, 70–230 mesh, Merck, Darmstadt, Germany) column chromatography (9 × 28 cm). The column was eluted with a gradient of dichloromethane/acetone, increasing the polarity of the eluting system with acetone, collecting volumes 350 mL, to obtain seven fractions; Fr-A (1.5 g, 95:5), FrB (0.8, 90:10), FrC (3.0 g, 80:20), FrD (2.5, 70:30), FrE (0.4, 60:40), FrF (1.3, 50:50,) and FrG (0.9, 0:100).

The FrC fraction (2.8 g) was fractionated on a chromatographic column (5 × 30 cm) with silica gel (90 g, 70-230 mesh, Merck). The column was eluted with a gradient of dichloromethane/acetone/methanol, increasing the polarity of the system by acetone and methanol, collecting volumes of 100 mL to give six fractions; FrC-1 (100:0:0), FrC-2 (95:5:0), FrC-3 (90:10:0), FrC-4 (80:20:0), FrC-5 (50:50:0), FrC-6 (50:50:50).

A yellow amorphous solid (54 mg) was obtained from the FRC-2 fraction; the structure of this compound was identified on the basis of 1D and 2D NMR techniques, and comparing with certain literature data [38]. It was identified as N-*trans*-feruloyltyramine (NTF), with a purity of 98%; mp 74–75 °C. UV (MeOH) nm (λ); 220, 293,318. ^1^H NMR (400 MHz, CD_3_OD); ^13^C NMR (100 MHz, CD_3_OD) (see Supplementary Information); FABMS *m/z* 314[M + H]^+^; HRFABMS *m/z* 314 [M+ H]^+^; calculated for C_18_H_19_NO_4_.

### 3.4. Standardization of Fractions with N-Trans-Feruloyltyramine by HPLC

The extract Ace-Sa and fractions (F1, F2 and F3) were analyzed by a HPLC technique, and a calibration curve was used with NFT; this allowed us to standardize the samples from *S.* *aristolochiaefolia*. The concentrations analyzed of compound 1 were: 2.77 mg/g for Ace-Sa, 26.34 mg/g for F1, 60.22 mg/g for F2 and 8.76 mg/g for F3. The method was carried out in a Waters system (Separation Module 2695, Milford, MA, USA) coupled to a diode array detector (2996) with a 190–600 nm detection range and operated Empower 1 software system [39]. Separations were performed in a Superspher RP-18 column (125 × 4 mm, 5 µm; Merck) employing a constant temperature of 25 °C during analyses. Mobile phase is composed by solvent A (H_2_O, TFA 0.1%) and B (CH_3_CN, TECSIQUIM, Toluca, Edo Mex, Mexico) gradient system with a flow of 1.0 mL/min during 28 min. The ratio mobile phase remained in the following way: A:B = 100:0 (0–1 min), 90:10 (2–4 min), 80:20 (5–7 min), 70:30 (8–14 min), 60:40 (15–18 min), 20:80 (19–22 min), 0:100 (23–26 min) and 100:0 (27–28 min).The sample injection volume was 20 µL an the detection wavelength was scanned at 190–400 nm.

### 3.5. Animals

Male C57/BL-6 mice (Harlan México, Mexico City, Distrito Federal, México) were used for all experiments, which were housed and maintained under the following laboratory conditions: 25 °C ± 3, a 12 h:12 h light/dark cycle (lights on at 7:00), free access to water and food, either normal diet (pellets from Harlan rodent lab diet) or high-caloric diet, depending on the experimental group concerned. All studies were carried out in accordance with the official Mexican regulations NOM-062-ZOO-1999 (Technical Specifications for Production, Care, and Use of Laboratory Animals). The experimental protocol was authorized by the Local Health Research Committee (IMSS, Register number: R-2010-1701-41).

### 3.6. Feeding the Mice with a High-Caloric Diet and a Normal Diet

A batch of 96 mice was divided into eight groups. Basal condition: received a regular diet (RD); mice with only high-caloric diet (HCD, control I). All other groups were subjected to chronic damage with HCD and angiotensin II (AGII, 0.1 mg/kg i.p.) by twenty weeks and then animals without any treatment represented the negative control group (control II). The experimental treatments for next groups were: acetone extract from *S. aristolochiifolia* (Ace-Sa) and F1, 25.0 mg/kg; F2, 10 mg/kg and F3, 25 mg/kg, all orally. The positive control group was administered with Pioglitazone (≥99% purity, Sigma-Adrich, St Louis, MO, USA) (10 mg/kg) and telmisartan (≥98% purity, Sigma-Adrich) (10 mg/kg). The different treatments were administered from week 10 to 20. During the trial period, the weight gain of each mouse was recorded weekly to establish the growth curve. At the end of the trial period, the following parameters were measured: body density, fasting glucose, insulin tolerance curve, triglycerides, blood pressure.

### 3.7. Determination of Body Density

Under surgical anesthesia (pentobarbital sodium 50 mg/kg ip, Pfizer, México City, Distrito Federal, México), the mice were immersed in a graduated volumetric device with 100 mL of water, keeping the nose of the rodents out of the water. The volume of water displaced is measured and recorded; the ratio between weight and displaced volume is calculated and this index corresponds to body density.

### 3.8. Determination of Fasting Glucose

To determine the glucose concentration of the animals exposed to different treatments, previously fasted for 12 h, a blood sample of 5 μL was obtained from the tail vein and the glucose was quantified (mg/dL) using a Bayer Contour^®^ TS glucometer.

### 3.9. Insulin Tolerance Curve

The insulin tolerance curve was traced at the end of the treatments. Five μL of blood from non‑fasting mice were obtained from the tail vein; the glucose was quantified (mg/dL) using a Bayer Contour TS glucometer. Subsequently, a dose of 0.1 U/kg of rapid insulin was applied (Humulin R, Lilly^®^, Indianapolis, IN, USA) via i.p. and the glucose was quantified in the manner already described, 15, 30, 60 and 120 min after the application. The response curve was constructed with the data obtained, and the area under the curve was calculated.

### 3.10. Determination of Plasmatic Triglycerides

The plasmatic concentration of triglycerides was determined in plasma from mice exposed to the different treatments by enzymatic colorimetric assay using the Wiener lab Color TG GPO/PAP AA, (Rosario, Santa Fe, Argentina) following the recommendations of the supplier. The plates were read in an ELISA reader (Awareness Technology Inc. [40]) model Stat Fax-2100, city, state abbrev if US, country) and the sample absorbance at 492 nm was recorded.

### 3.11. Euthanasia

After 20 weeks of treatment, the mice were deeply anesthetized i.p. with pentobarbital sodium at a dose of 80 mg/kg and blood samples were quickly obtained from the infraorbital sinus of each mouse. The plasma was obtained by centrifugation (2500 rpm for 15 min) at room temperature; then the samples were stored individually at −10 °C for the various determinations.

### 3.12. Quantification of Cytokines from Mouse Kidney and Adipose Tissue

The kidney and adipose tissue of mice was transferred to 15-mL tubes, placed on dry ice, and suspended in phenyl methyl sulfonyl fluoride (PMSF; Sigma-Aldrich) 0.1 mM in PBS (1 mL/200 mg of tissue). Samples were homogenized (Potter-Elvehjem homogenizer Thomas Scientific, Swedesboro, NJ, USA) and then centrifuged for 10 min at 14,000 rpm at 4 °C. The supernatants were transferred to 1.5-mL Eppendorf tubes and were ready for analysis. IL-1β, IL-6, IL-10 and TNFα were measured using ELISA BD OptEIA™ (San Jose, CA, USA), according to the manufacturer’s instructions. Concentrations for each cytokine were calculated from calibration curves using individual recombinant proteins as standards, also according to the manufacturer’s instructions. Density (OD) readings were performed at 30 and 60 min of incubation at 450 nm. The value of the concentration of pro-inflammatory cytokines (IL-1β, IL-6 and TNFα) was divided by the value of the concentration of anti-inflammatory cytokine (IL-10). Finally, the resulting index was compared with the baseline value.

### 3.13. Blood Pressure Measurement

The mice exposed to the different treatments were anesthetized (pentobarbital 50 mg/kg i.p., Pfizer) and placed in a LETICA Storage Pressure Meter, LE 5002, (Biopac System MP150, Goleta, CA, USA [41]). Ten measurements were recorded for each mouse and an average was obtained to report the PAS and PAD of each case.

### 3.14. Statistical Analyses

The results are expressed as the mean ± SEM and were analyzed using the analysis of variance (ANOVA) with a Tukey *post-test* (* *p* < 0.05).

## 4. Conclusions

It is concluded that the extract and fractions with NTF are options in the treatment of the symptomatology associated with the MS.

## Supplementary Materials

Supplementary materials can be accessed at: http://www.mdpi.com/1420-3049/19/8/11366/s1.
