# Supplementary material for: Hypoglycemic and Hypotensive Activity of a Root Extract of Smilax aristolochiifolia, Standardized on N-trans-Feruloyl-Tyramine
Source: Molecules. 2014 Jul 31;19(8):11366–84. doi: 10.3390/molecules190811366 (PMC6271314; doi:10.3390/molecules190811366)
Supplement: Supplementary file 1 [file molecules-19-11366-s001.pdf]

Supplementary Materials

Table S1. Data of ^{1}H and ^{13}C-NMR from the compound n-trans-feruloyltyramine at 400 MHz for proton and 100 MHz for carbon (in methanol).

No.	δ_c	$\delta_{H(J\ in\ Hz)}$	δ_C [39]
1	128.39		129.77
2	111.6	7.1 (d, 2)	114.99
3	149.3		147.32
4	149.9		147.81
5	116.5	6.79 (d, 8.2)	116.71
6	123.33	7.0 (dd, 2, 8.2)	110.31
7	142.1	7.43 (d, 15.6)	122.70
8	118.8	6.4 (d, 15.6)	140.72
9	169.28		167.12
-OCH_3	56.51	3.87 (s)	55.56
1'	131.41		127.0
2'	130.85	7.05 (d, 6.8)	115.17
3'	116.39	6.72 (d, 7.2)	129.40
4'	157		155.16
5'	116.39	6.72 (d, 7.2)	129.40
6'	130.85	7.05 (d, 6.8)	115.17
7'	35.92	2.75 (dd, 7.1, 7.5)	34.39
8'	42.66	3.46 (dd, 7.2, 6.5)	40.56

Figure S1. ^{1}H-NMR (CD_3OD, 400 MHz) Compound 1.

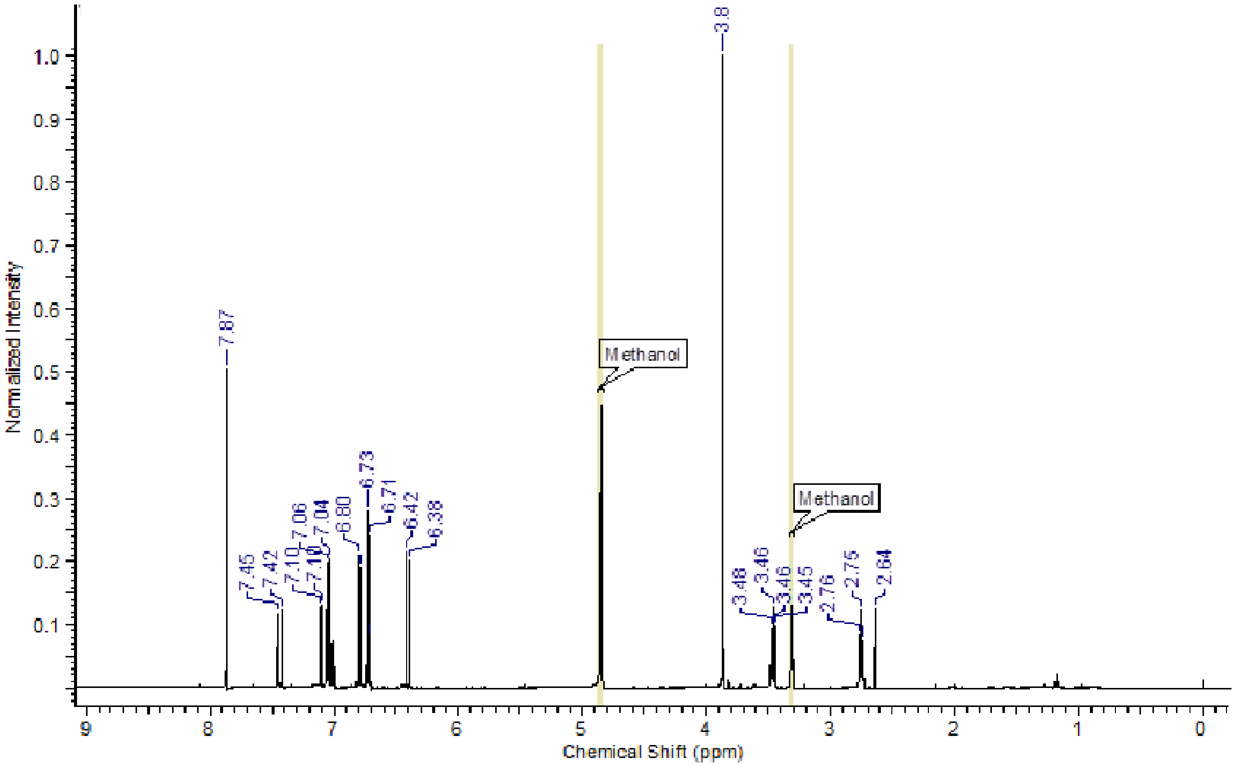

Figure S2. ^{13}C-NMR (CD_3OD, 400 MHz) Compound 1.

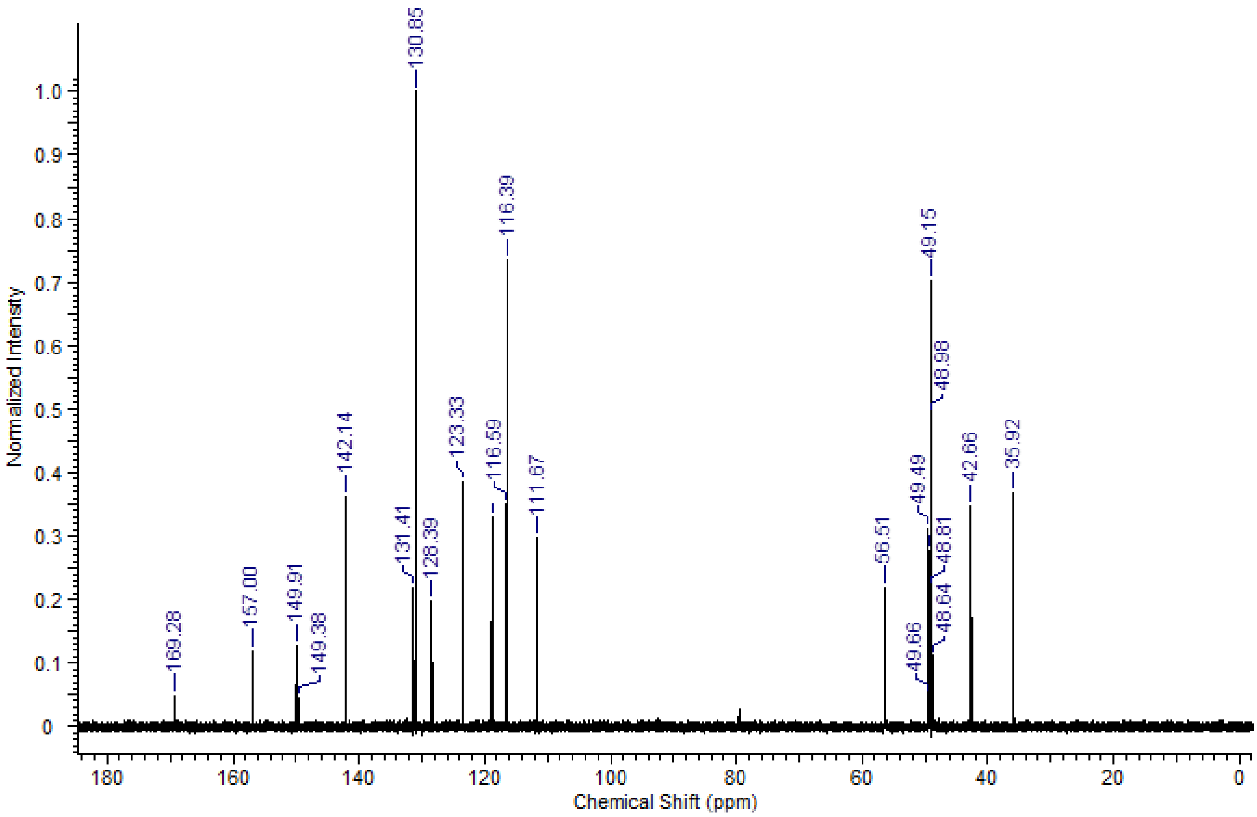

Figure S3. ^{1}H–^{1}H COSY (CD_3OD, 400 MHz) Compound 1.

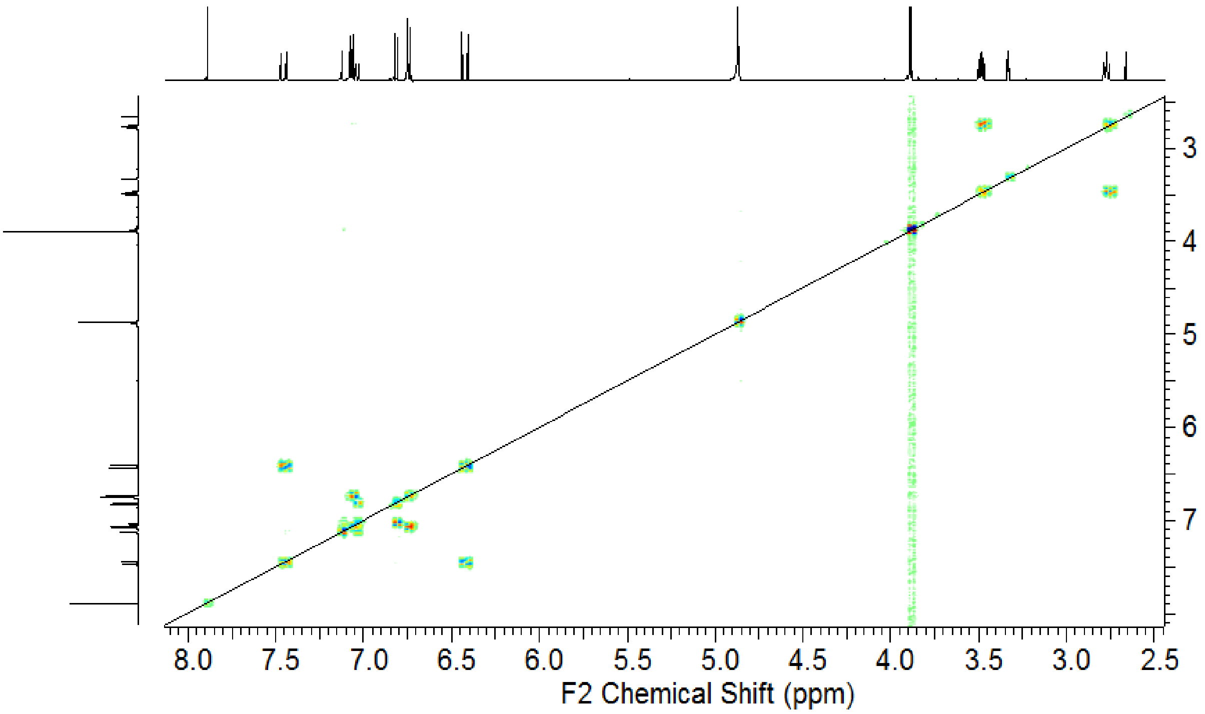

Figure S4. HSQC (CD_3OD, 400 MHz) Compound 1.

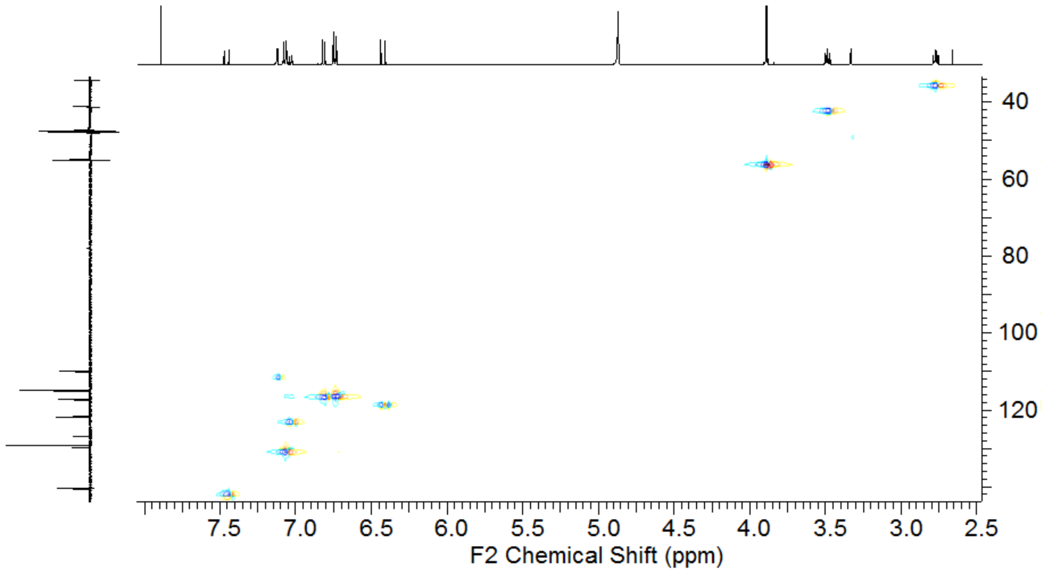

Figure S5. HMBC (CD_3OD, 400 MHz) Compound 1.

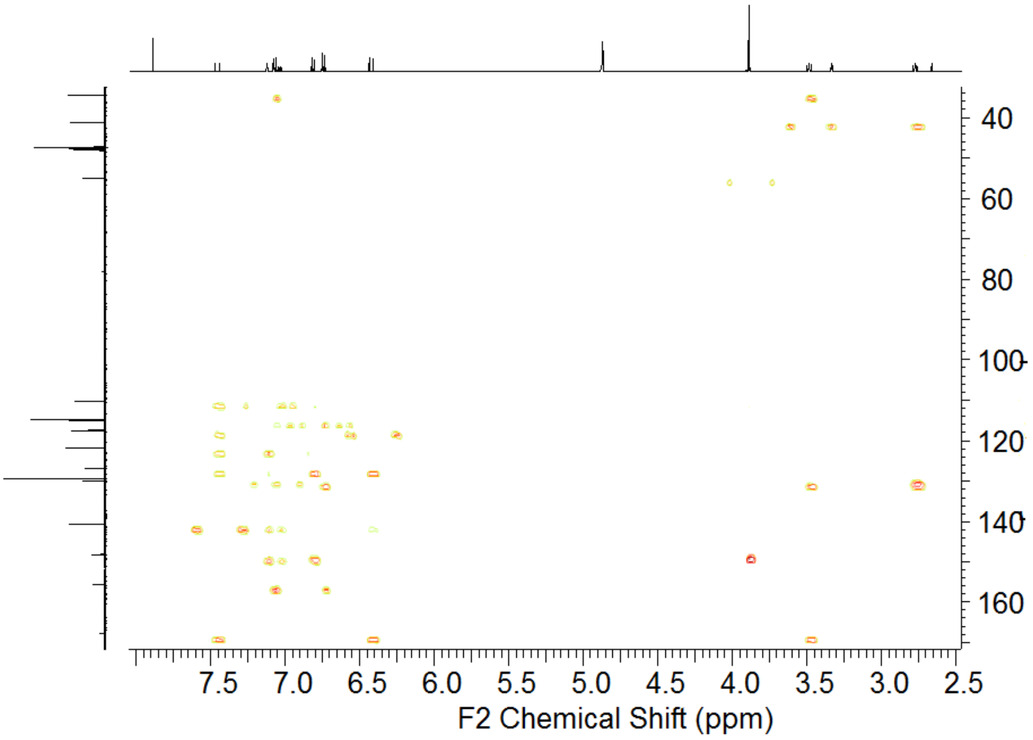

Figure S6. IR spectra Compound 1.

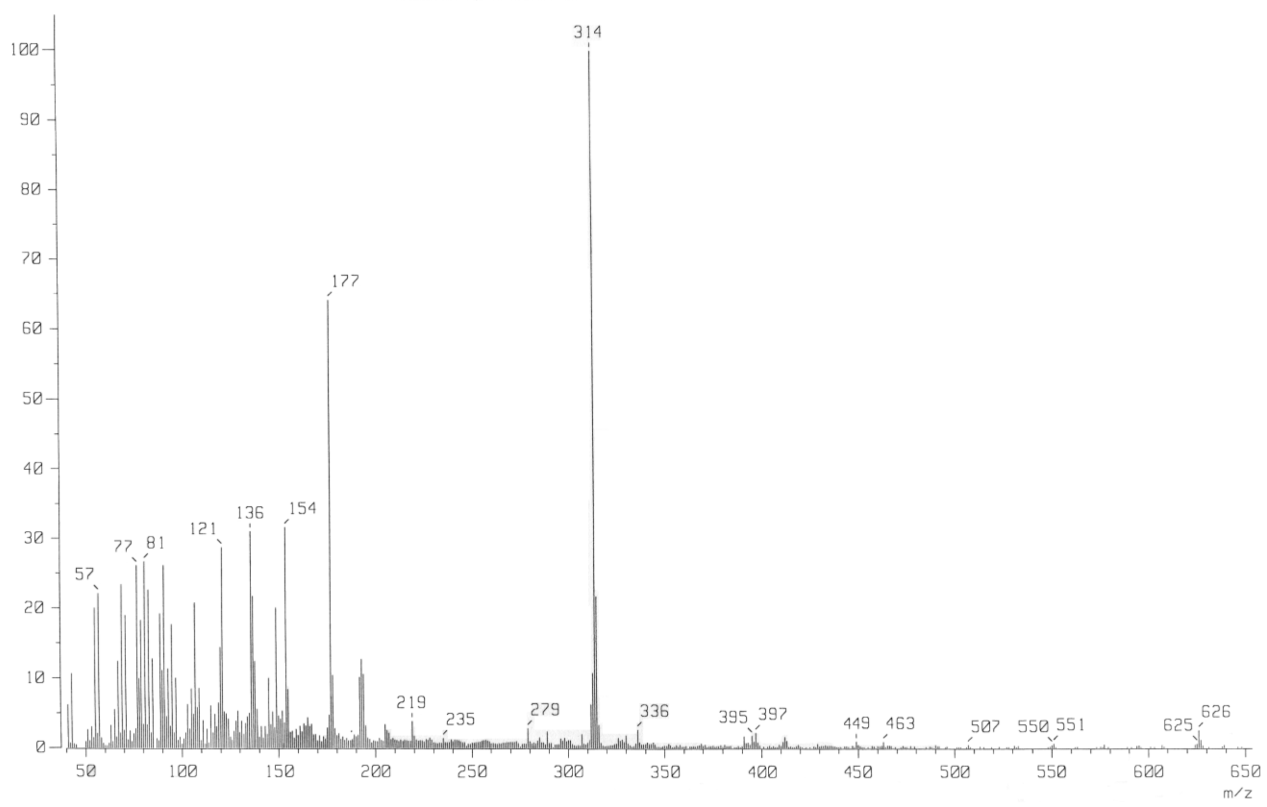

Figure S7. Calibration curve NFT.

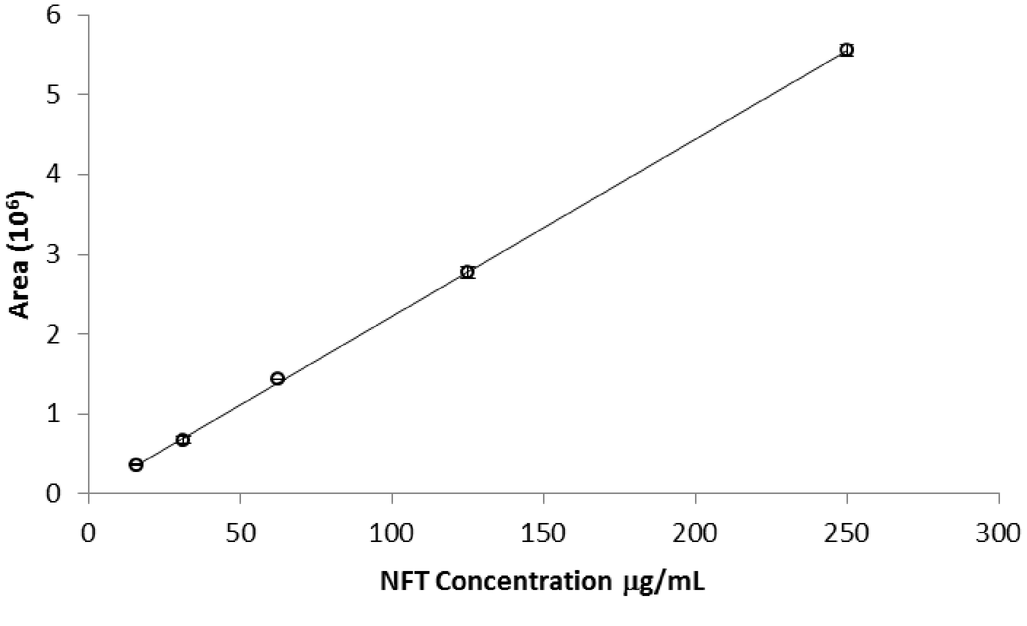

Table S2. Parameters validation of HPLC method for the quantification of NTF.

Parameter	Value	Unit
LOD	4.09	µg/mL
LOQ	11.28	µg/mL
Correlation	0.9998	
Low concentration (5 µg/mL)	4.84 ± 0.72 (−3.2)	µg/mL (Bias%*)
Medium concentration (50 µg/mL)	49.37 ± 0.81 (−1.3)	µg/mL (Bias%*)
High concentration (125 µg/mL)	127.14 ± 1.15 (1.7)	µg/mL (Bias%*)

* (% Bias) = [(Cobs − Cnom)/Cnom] × 100.

Table S3. NFT relative concentration of the area under the curve the chromatographic peaks in each fraction.

Retention Time (min)	SaAce	F1	F2	F3
7.183	2.49		2.29	
8.339				11.40
8.766		7.84		4.16
9.304		4.59	13.74	16.55
10.186 (NTF)	2.77	2.63	60.22	8.76
10.527	14.99	39.87	14.29	13.70
11.706	3.73	20.42	6.63	
15.428	10.46	8.54	9.44	
20.191	9.29	15.72	19.48	
Doses NTF µg/kg	69	66	600	219
